# The Mechanisms and the Applications of Antibacterial Polymers in Surface Modification on Medical Devices

**DOI:** 10.3389/fbioe.2020.00910

**Published:** 2020-11-11

**Authors:** Haofeng Qiu, Zhangyong Si, Yang Luo, Peipei Feng, Xujin Wu, Wenjia Hou, Yabin Zhu, Mary B. Chan-Park, Long Xu, Dongmei Huang

**Affiliations:** ^1^School of Medicine, Ningbo University, Ningbo, China; ^2^School of Chemical and Biomedical Engineering, Nanyang Technological University, Singapore, Singapore; ^3^Faculty of Materials Science and Chemical Engineering, Ningbo University, Ningbo, China; ^4^Ningbo Baoting Biotechnology Co., Ltd., Ningbo, China

**Keywords:** antibacterial polymer, mechanism, surface coating, medical device, antibacterial effect

## Abstract

Medical device contamination caused by microbial pathogens such as bacteria and fungi has posed a severe threat to the patients’ health in hospitals. Due to the increasing resistance of pathogens to antibiotics, the efficacy of traditional antibiotics treatment is gradually decreasing for the infection treatment. Therefore, it is urgent to develop new antibacterial drugs to meet clinical or civilian needs. Antibacterial polymers have attracted the interests of researchers due to their unique bactericidal mechanism and excellent antibacterial effect. This article reviews the mechanism and advantages of antimicrobial polymers and the consideration for their translation. Their applications and advances in medical device surface coating were also reviewed. The information will provide a valuable reference to design and develop antibacterial devices that are resistant to pathogenic infections.

## Introduction

Various medical devices, such as intravascular catheters, heart valves, orthopedic implants, dental implants, and contact lenses are often used clinically to treat diseases (Francolini et al., 2015). However, there are often a large number of pathogenic microorganisms such as bacteria and fungi in the nosocomial environment that may injure patients secondly due to microbial infection, which sometimes even results in serious complications like sepsis and induces high morbidity and mortality. “China Health Statistics Yearbook 2019” published by National Health Commission stated that 29.3% patients with foodborne diseases were infected by microorganisms. In the United States of America, the annual cost responding to infections with drug-resistant bacteria had reached $21–34 billion (Spellberg et al., 2011). The report about the global prevalence of ESKAPE (*Enterococcus faecium*, *Staphylococcus aureus*, *Klebsiella pneumoniae*, *Acinetobacter baumannii*, *Pseudomonas aeruginosa*, and *Enterobacter* spp.) pathogens stated that ESKAPE had developed resistance to almost all known antimicrobials due to their particular antimicrobial resistant characteristics (Kamaruzzaman et al., 2019). The consequence of economic losses in some countries had reached 0.4–1.6% value of GDP (Smith et al., 2005). The infections by bacteria, especially by antibiotic-resistant bacteria, caused serious harm to human beings. And, there are few medicines till now are quite effective against these antibiotic-resistant bacteria.

On the other hand, there are always biomolecules like bacteria, cells, proteins, and polysaccharides, *etc*., adhered on device surfaces, as the device surfaces provide commensal benefits for them. These biological substances will form a biofilm on device surface that protects bacteria from being killed. Generally, biofilm is 100–1000 times more resistant than planktonic bacteria (Mishra et al., 2015). Antibiotic resistance is now a leading threat to human health because patients have to take more amounts of drugs to tackle the infection. The emergence of multi-drug-resistant bacteria that contaminate medical equipment and devices has put patients at considerable risk of being infected repeatedly, which is harmful and even lethal (Orapiriyakul et al., 2018).

In order to prevent the contamination from microorganisms, making antimicrobial coating on medical devices is the most straightforward method (Francolini et al., 2017). On the one hand, it prevents secondary bacterial contamination to patients; on the other hand, it is also favorable to prevent the increase of drug-resistant pathogens through reducing medicine administration, which further cuts down the pollution to the environment due to reduced antibiotics leaching (Riga et al., 2017). Therefore, it is urgent to develop antimicrobial materials with function as antibiotics and without drug resistance, so that microbial resistant medical instruments and equipment can be provided to hospitals and patients. Polymeric materials, such as cationic polymers, are a class of environmentally friendly antibacterial agents that are not prone to cause drug resistance, because they mainly kill bacteria by damaging the cell membrane. In addition, artificial polymers can be designed and synthesized to meet medical requirements but not restricted by the device shape. Thus, antibacterial polymers will be a promising alternative to antibiotics. Therefore, in this article we will review the mechanism, advantages and their potential clinical applications of a variety of antibacterial polymers, which will bridge the gap of scientific research and the practical or potential applications. It is believed that the information proposed in this article will provide a valuable reference in designing and developing antibacterial medical devices in future.

## Mechanism of Antibacterial Effect

To clarify the antibacterial mechanism, we must understand the structural characteristics of bacteria at first. The biologic structure of bacteria is similar to that of cells. It is mainly composed of cell wall, cell membrane and intracellular components. Some bacteria have special structures like capsule, flagella, pili, and spores (Silhavy et al., 2010). As early as 1884, Hans Christian Gram, a Danish bacteriologist, invented a method to classify bacteria known as Gram staining. According to the reaction with Hexamethyl-p-phenylenediamine chloride, a crystal violet dye, he divided the bacteria into two categories: Gram-positive and Gram-negative bacteria. This method has been widely used in screening or identifying organisms since then. Gram-positive bacteria have a thick network of cell walls made of peptidoglycan, which retains the crystal violet dye. Thus, the bacteria display purple. Instead, Gram-negative bacteria have too thin cell walls to retain crystal violet dye. Thus, the bacteria are not stained.

Both Gram-positive and Gram-negative bacteria have cytoplasmic membranes, which are phospholipid bilayers containing functional proteins. The phospholipid bilayers include anionic lipids of cardiolipin (CL), phosphatidylglycerol (PG), and phosphatidylserine (PS), which is vastly different from mammalian cell membranes that are rich in zwitterionic lipids of phosphatidylcholine (PC), phosphatidylethanolamine (PE), and cholesterol. Therefore, bacteria are more negatively charged compared to mammalian cells in structure (Li et al., 2012). This feature has been used by scientists to explore new antibacterial medicines that can selectively kill bacteria while they do not display toxicity to mammalian cells. This is the main concept recognized by researchers to distinguish bacteria from mammalian cells.

Except for the cytoplasmic membrane, the Gram-negative bacteria have additional outer membranes which are composed of anionic lipopolysaccharide (LPS) molecules crosslinked by the ionic bridges between the phosphate groups and divalent ions. Thus, the outer membranes of Gram-negative bacteria are very stable and function as permeabilizing barriers to most large hydrophobicity molecules.

### Targeting the Bacterial Membranes

The cytoplasmic membrane is a valid target to eradicate bacteria. The damaged membrane is usually very difficult to renew. Many cationic polymers combat bacteria via electrostatic attraction to the cell membrane, followed by the hydrophobic insertion into lipid tails, resulting in membrane lysis. Cationic polymers generally contain positively charged functional groups like amine and guanidine in the molecules, while bacterial membranes are negatively charged under normal status. Through the electrostatic force between positive and negative charges, the cationic materials are attracted to the anionic bacterial membrane, and further penetrate into or become part of the bacterial cytoplasm; they can also insert into the phospholipid bilayer, resulting in the membrane broken. It is worth noticing that not all the cationic molecules will be toxic to bacteria. The molecules will be harmful to the negatively charged bacteria membrane when the cationicity reaches a certain amount to achieve the multivalence effect. Most of the amphiphilic polymers consisting of cationic and hydrophobic residues killed the bacteria via the membrane lysis mechanism (Chin et al., 2013; Hoque et al., 2015).

Several models have been proposed by researchers to explain the membrane lysis mechanism (Wimley, 2010): “Carpet” model, which explains that the antimicrobial agents destroy the phospholipid structure of bacterial membranes at multiple sites at the same time to induce many irreparable holes in membranes; “Toroidal-pore” model, which illustrates that antibacterial molecules can insert spirally into the bacterial membrane to form circular holes in the membrane surface; “Barrel” model, which tells that the antibacterial molecules screw themselves into bundle-like spiral molecules in the membrane. The hydrophobic region of the molecules parallels to the membrane lipids while the hydrophilic ends will form pores in the cytoplasmic membrane. In all these cases, they eventually lead to bacteria death via membrane lysis (Brogden, 2005).

As the Gram-negative bacteria have an additional outer membrane that is a barrier to the large hydrophobic molecules, most of the cationic polymers have less potency against Gram-negative bacteria compared to that of Gram-positive bacteria (Silhavy et al., 2010; Cox and Wright, 2013). Instead of targeting the cytoplasmic membrane, the outer membrane of Gram-negative bacteria may also be the active targets for cationic polymers. Colistin, the last-resort antibiotic against Gram-negative bacteria, initially interacted with the negatively charged LPS in the bacterial outer membrane. As colistin bound to LPS much stronger than the divalent cations Mg^2+^ or Ca^2+^, which otherwise tightly hold the LPS molecules together, it competitively replaced these divalent ions to weaken and release the LPS molecules to enable the colistin to reach the bacterial cytoplasmic membrane resulting in bacterial lysis (Sabnis et al., 2018).

However, the cationic polymers targeting bacterial membrane usually produce a low selectivity, which might bring the cationic polymers failed in clinical trials (Gordon et al., 2005), because the amphiphilic structure of cationic and hydrophobic polymers may engage in hydrophobic interaction with mammalian cell cytoplasmic membranes, resulting in cytotoxicity. Thus, the new mechanisms of cationic polymer actions are highly sought for.

The cationic peptidomimetic murepavadin will selectively combat Gram-negative bacteria *P. aeruginosa* by targeting the essential protein LptD (Srinivas et al., 2010), a translocator of LPS molecules, in the outer membrane of Gram-negative bacteria. As the mammalian cell membranes do not include LptD, the off-target toxicity in mammalian cells may be mitigated. In addition to LptD, six more Lpt proteins are involved in translocating LPS synthesized in the cytoplasmic membrane to the outer leaflet of the outer membrane via constructing a macromolecular complex connecting the cytoplasmic membrane and the outer membrane. Cationic antimicrobial peptide (AMP) thanatin targets not only LptD in the outer membrane, but also targets the periplasmic protein Lpt A (Vetterli et al., 2018). The dual targets of thanatin may contribute to its low possibility of resistance evolution. Another essential protein in the outer membrane is BamA, a chaperone and translocator that folds and inserts β-barrel proteins into the outer membrane. Darobactin selectively killed Gram-negative bacteria by targeting BamA to prevent its functions (Imai et al., 2019). However, due to the specific protein target of darobactin, the resistant mutants occurred after a few generations. The progress in this area leads to another interesting cationic chimeric peptidomimetics that target both the LPS and BamA (Luther et al., 2019). As the multiple targets, the frequency of resistant mutants to them is less than 10^–10^. Except for the essential proteins in outer membrane, the cytoplasmic membrane contains some potential protein targets too; the cationic macrocyclic peptides G0775 target the LepB (Smith et al., 2018), an essential protease in charge of cleavage of the signal peptides. G0775 has broad-spectrum antimicrobial activities against both Gram-positive and Gram-negative bacteria. The electron transport chain (ETC) in bacterial cytoplasm in charge of a series of essential cellular processes (Hurdle et al., 2011), such as bacterial respiration, antitoxins, nutrient uptake, *etc*., represents another interesting target for combating bacteria. Lysocin E was the firstly reported cationic peptide that kills bacteria through targeting menaquinone (Hamamoto et al., 2015; Itoh et al., 2019), the shutter in the ETC. The damage of the ETC may generate reactive oxygen species (ROS) that can further cause impairment to many cellular molecules, such as DNA and proteins (Painter et al., 2017). Cationic star polymer SNAPPs killed bacteria involving in the generation of ROS (Lam et al., 2016).

### Inhibition of Cell Wall Synthesis

The cell wall structure mainly consisting of peptidoglycans provides bacteria with mechanical support to maintain morphology. These peptidoglycan layers are made of N-acetylglucosamine and N-acetylmuramic acid connecting by β-1,4 glyosidic bonds. The occurrence of crosslinking among glycans via peptide chains forms stable wall networks. The lipid II is the essential precursor for the biosynthesis of peptidoglycan layers (Breukink and de Kruijff, 2006; Malin and de Leeuw, 2019), which could be the targets of cationic polymers to inhibit the cell wall synthesis. Cationic peptide teixobactin killed Gram-positive bacteria *S. aureus* via targeting the lipid II to prevent the cell wall synthesis (Ling et al., 2015). Lipid II is also the targets of many other cationic peptides, such as plectasin (Schneider et al., 2010), nisin (Scherer et al., 2015), telavancin (Saravolatz et al., 2009), teicoplanin (Somma et al., 1984), *etc*. As the lipid II only exists in bacteria, the non-specific toxicity to the mammalian cells may be decreased. Daptomycin, the last-resort of antibiotic against drug-resistant Gram-positive bacteria approved, was reported to kill bacteria via membrane pore formation. Nevertheless, Hamoen et al. found recently that daptomycin only caused the decrease in membrane potential without membrane pore formation (Müller et al., 2016). The key action of daptomycin is blocking the cell wall synthesis by the removal of lipid II synthesis proteins. Lipid II is synthesized in the bacterial cytosol and transported by lipid shutters, undecaprenol phosphate, and flippase. Therefore, molecules that are able to target the lipid II shutters may also inhibit cell wall synthesis. Indeed, cationic peptide friulimicins and humimycins prevent the cell wall synthesis of Gram-positive bacteria by engaging in blocking undecaprenol phosphate and flippases (Liu et al., 2019), respectively. As the Gram-negative bacteria have much thinker peptidoglycan layers, which are covered by the outer membrane barrier, thus most of the large molecule weight polymers only inhibit the cell wall synthesis in Gram-positive bacteria.

### Targeting the Intracellular Molecules

In the case of cationic polymers that can cross the outer membrane and cytoplasmic membrane barriers, they may accumulate in the cytosol and interrupt the intracellular machineries. DNA is the molecular basis for bacterial replication. The damage to DNA will adversely affect its synthesis and replication of bacteria. Cationic polymer polyhexamethylene biguanide (PHMB) can enter into bacteria to inhibit cell division and condense bacterial chromosomes via direct interaction with DNA (Chindera et al., 2016). After binding to DNA, PHMB will condense the DNA to form the nanoparticles, which may result from the electrostatic interaction between the cationic PHMB and anionic DNA molecules. A similar mechanism was proposed for the biodegradable guanidinium-functionalized polycarbonates (Chin et al., 2018), which eradicate bacteria without membrane morphological changes. Interestingly, both the PHMB and guanidinium-functionalized polymers have the guanidinium functional groups, which may contribute to the cell-penetrating effect of these cationic polymers. Recently, Liu et al. reported that cationic poly(2-oxazoline) selectively killed *S. aureus* (Zhou et al., 2020), engaging in strong interaction with DNA at low concentration, which results in producing ROS to damage the membrane and kill the bacteria. Duarte et al. isolated an AMP ruminococcin C1 (RumC1) from human gut symbiont (Chiumento et al., 2019), which displayed similar phenotype as metronidazole that acts through inhibition of nucleic acid synthesis. Thus, the authors proposed that RumC1 combats bacteria through most likely inhibition of nucleic acid synthesis in a metronidazole-like manner.

Protein synthesis occurs in the ribosome of bacteria. The ribosome is a possible target for cationic polymers to work. Steitz et al. found that bovine peptide bactenecin Bac7_1–35_ preferentially interacted with the 70S ribosome (Gagnon et al., 2016) to inhibit the initiation of translation, resulting in protein synthesis inhibition. Oncocin, apidaecin, and its derivative Api137 are another three proline-rich AMPs that can inhibit protein synthesis via target the bacterial ribosome (Krizsan et al., 2014; Florin et al., 2017).

### Immunomodulatory

In addition to the direct eradication of bacteria based on the mechanism discussed above, some cationic polymers act as immunomodulators to combat bacterial infections (Mookherjee et al., 2020), which include recruiting the immune cells, such as neutrophils, macrophages, and T cells, to the infection sites to promote the bacterial clearance, enhancing the neutrophil function, suppressing the release of pro-inflammatory cytokines and facilitating the induction of anti-inflammatory cytokines, promoting phagocytosis, *etc*. Hancock and Sahl (2006) first reported an innate defense-regulator peptide (IDR-1) that itself did not have direct antimicrobial efficacy (Scott et al., 2007), but it can activate mitogen-activated protein kinase and other signaling pathways, enhancing the level of monocyte chemokines and suppressing the pathogen-associated signature molecules to protect the mice from infection by both Gram-negative bacteria and Gram-positive bacteria. Cationic AMP LL-37 can clear the bacterial infection via facilitating neutrophil response (Beaumont et al., 2014). Although the *in vitro* action of cationic star polymer SNAPPs includes mechanisms like membrane disruption, facilitating ions to cross the cytoplasmic membrane, generation of ROS (Lam et al., 2016), its *in vivo* antimicrobial activity actually engaged in the recruitment of neutrophils. Compared to the direct killing of cationic polymers, which are often affected by protein fouling, salt concentration, *etc*., the indirect killing of cationic polymers based on modulating immune response represents a promising strategy to develop therapeutic antimicrobial agents.

**FIGURE 1:**
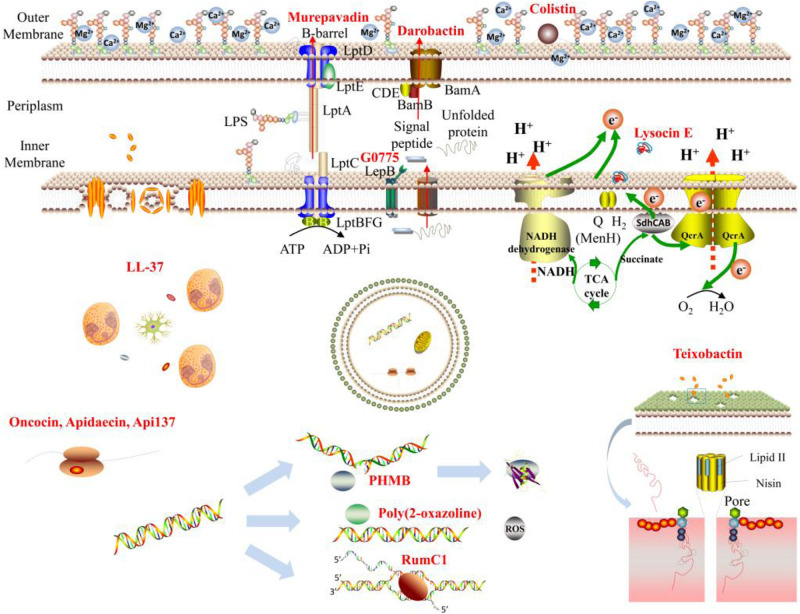
Schematic diagram of antibacterial mechanisms.

All these antibacterial mechanisms were summarized as diagram in Figure 1.

## Classification and Properties of Antibacterial Materials

The repeated application of traditional antibiotics may cause genetic mutations of microorganism, resulting in the drug-resistant microorganisms (Vasilev et al., 2014). Four types of antibiotic resistance mechanisms were reported (Bahman et al., 2016): (1) bacteria can block antibiotics through reducing the outer membrane permeability; (2) bacteria can cut down or eliminate the medicine effect through adjusting self-biology if antibiotics enter into the cells; (3) bacteria will produce corresponding enzymes to degrade antibiotics; (4) bacteria alter themselves histology to change the target site that antibiotics act on. Differently, cationic polymers are able to overcome the defect of these drug resistance pathways in bacteria. Moreover, artificial cationic polymers have the feature of synthetic diversities. Many of cationic polymers have been evaluated to be antimicrobial candidates. Importantly, antibacterial effect and cell toxicity of them are capable of being regulated artificially through adjusting the molecular structure, molecular weight, charge density, and amphiphilic balance, *etc*., to finally achieve the material properties with the excellent antimicrobial effect and low toxicity to cells or tissues (Jaeger et al., 2010). Therefore, cationic antimicrobial polymers are considered to be the last frontier of antibiotic development. The follows review the preparation, classification and performance of antibacterial polymers.

### Cationic Polymers

Polypyridine (PPy) derivatives are a class of heterocyclic cationic polymers containing quaternary ammonium groups. Li and Shen (2000) synthesized copolymers of 4-vinylpyridine (VP), styrene and divinylbenzene (DVB), followed by quaternization of halogenated hydrocarbons. This copolymer possessed a good bactericidal effect on *Escherichia coli*, but has poor biocompatibility to mammalian cells. In order to overcome this limitation, hydrophilic polypyridine was copolymerized with 2-hydroxyethyl methacrylate (HEMA) and poly (ethylene glycol) methyl ether methacrylate (PEGMA) as the starting materials, followed by reaction with N-hexyl poly (4-vinylpyridine). The synthetic hydrophilic polypyridine copolymer not only displayed significant improvement in biocompatibility, but also increased the antibacterial performance as high as 20 times than that of quaternized poly (vinylpyridine) alone (Allison et al., 2007; Sellenet et al., 2007).

Poly ionic liquids are a group of polyelectrolytes that consist of both anion and cationic groups on the repeating units of polymeric backbones (Zhang et al., 2020). The positive residues can interact with the negative bacterial cell membrane via electrostatic interaction, resulting in disrupting the integrity of bacterial cell membrane. Therefore, poly ionic liquids may be a novel type of effective antibacterial materials. Poly-imidazolium polymers are a series of poly ionic liquids that are readily prepared via either step-by-step synthesis or one-pot polycondensation reactions. Liu et al. (2012) firstly prepared a series of main-chain imidazolium polymers via step-by-step coupling reactions (Liu et al., 2012). Main-chain imidazolium oligomer (IBN-1) with 6 imidazolium repeat units displayed the highest potency against Gram-negative bacteria *E. coli*, *P. aeruginosa*, *K. pneumoniae*, *A. baumannii*, *Enterobacter cloacae* and Gram-positive bacteria of Methicillin-resistant *S. aureus* (MRSA), Vancomycin-resistant enterococcus (VRE) with the MICs in the range of 1.5–31.25 μg/ml. Although the authors reported that IBN-1 was almost non-hemolytic at a concentration of 5000 μg/mL, it was found to be highly toxic to mammalian fibroblast cells. In the following studies, the authors prepared another main-chain imidazolium polymer PIM-45 via poly-condensation reactions (Liu et al., 2013), which are effective against both planktonic fungi and fungal biofilm without hemolysis. However, it was still toxic to mammalian fibroblasts. To overcome the issue of bio-toxicity, the researchers recently fabricated a series of biodegradable imidazolium polymers via incorporating pH-degradable linkers into the polymer chains (Yuan et al., 2019). For example, IBN-HC8 with hemiaminal as the degradable linker exhibited good antimicrobial potency against Gram-positive *S. aureus* and Gram-negative *E. coli* as its parental material, IBN-C8, possessed the good biocompatibility to human primary dermal fibroblasts. Yuan et al. detected the antimicrobial potency of a series of cationic polymers with imidazolium rings in the main-chain or side-chain (Guo et al., 2018). Interestingly, they found that the antimicrobial potency of main-chain imidazolium polymers was much superior to the side-chain imidazolium polymers. The results provide insight for designing antimicrobial agents with high potency and low toxicity.

Guanidine-functionalized polymers are able to destroy the biological structure of bacteria by extracting calcium ion from the bacterial outer membrane and disrupt the cytoplasmic membrane (Santos et al., 2012). As calcium ions are crosslink agents for the LPS in outer membrane, the lack of them will increase the outer membrane permeability. The guanidine-functionalized polymers subsequently cross the outer membrane to interact with the cytoplasmic membrane via electrostatic interaction, which finally results in lysis of cytoplasmic membrane. Therefore, they have good antibacterial capabilities and thus been widely used as cationic antibacterial materials. Chin et al. (2018) prepared a series of guanidine-based polycarbonates (Chin et al., 2018). These materials exhibited excellent antibacterial properties and good selectivity; the therapeutic index [bactericidal concentration (LD_50_)/lethal concentration (ED_50_)] is 1473, in the experiments of *P. aeruginosa* lung infection of mice and perforation-induced polybacterial peritonitis. Their bactericidal effects were also investigated with mice infected by *Enterococcu*s, *S. aureus*, *K. pneumonia*, *A. baumannii*, and *Enterobacter* species (commonly known as ESKAPE bacteria). More importantly, these guanidine-based polycarbonates maintained the bactericidal function after repeated treatment for one month, indicating that the bacteria did not exhibit drug resistance to them, which is much better than antibiotics in this regard.

Conjugated oligomers (COEs) are another class of antibacterial materials with good bactericidal function. COEs are composed of hydrophobic and cationic building blocks in the backbone. This hydrophobic components will insert and break the bacterial cell membrane, aiming at killing bacteria (Liu et al., 2012; Bazan et al., 2018).

Dendritic polyethylene imine (PEI) contains both hydrophobic polyethylene main chain and imine group at the terminus. It has a high positive charge density (Davide et al., 2013). But the high charge density of PEI is toxic to animal cells. In order to achieve the balanced antibacterial activity and biocompatibility, literature (Paula et al., 2008) reported that the content and proportion of the molecular chain and functional groups, such as quaternary ammonium groups, alkyl chains with different lengths, allyl, and benzyl groups, *etc.*, shall be adjusted to a proper balance. The results showed that lower molecular weight usually has poorer bactericidal function, but larger molecular weight was more toxic to animal cells.

Antimicrobial peptides, basic polypeptides polymerized from 20∼60 amino acid residues, have been much studied by researchers due to their broad-spectrum antibacterial activity. They can be isolated from bacteria or animal tissues, or can be artificially synthesized (Gabriel et al., 2007). Compared with mammalian cells, the bacterial cell membrane is more negatively charged due to the accumulation of negatively charged LPS in Gram-negative bacteria or teichoic acid in Gram-positive bacteria. That’s why bacteria have a stronger electrostatic attraction to cations than mammalian cells do. Therefore, the amphiphilic AMPs can work to attach the bacterial cell membrane through positively charged groups while their hydrophobic bulk insert into the bacterial phospholipid bilayer to induce leakage or lysis of bacterial cell membrane (Kadurugamuwa and Beveridge, 1999).

However, AMPs are very difficult to produce because extracting from organisms is high cost and low yield. Some researchers have synthesized some AMPs by N-carboxy hydride (NCA) ring-opening polymerization (Wong et al., 2016; Wu et al., 2018). A series of star polymers were synthesized from NCA polymerization of lysine and leucine to mimic the natural AMPs. The results of *in vivo* tests in mice model verified that these star polymers had good bactericidal activity and good biocompatibility (Lam et al., 2016). Chan group polymerized with NCA and chitosan to simulate the peptidoglycan component of the bacterial cell wall (Li et al., 2012; Hou et al., 2017). On these bases, they designed a variety of glycosylated cationic chitosan-based polymers, whose antibacterial activity and selectivity can be adjusted to meet application requirements through changing the glycosyl composition and cationic residues (Pranantyo et al., 2017; Xu et al., 2019). For example, glucosamine-functionalized star polymers (S-GSA 25) showed high bactericidal activity against Gram-positive bacteria while maintained good biocompatibility (Wong et al., 2016).

As the natural AMPs are sensitive to the host enzymes that may render the AMPs ineffective during *in vivo* treatments (Kamaruzzaman et al., 2019). Therefore, AMPs memetics are widely investigated (Kamaruzzaman et al., 2019). Amongst them, β-peptides are one of the most promising candidates that have been widely studied as cationic antimicrobial peptidomimetics due to their structural similarity to natural α-peptides except for more resistant to host-enzyme degradation. Gellman et al. firstly prepared a non-hemolytic antimicrobial β-oligomer (β-17) *via* solid-phase synthesis. β-17 showed a comparable antimicrobial potency to natural AMP magainin against *E. coli* (MIC = 6.3 μg/mL), *Bacillus subtilis* (MIC = 1.6 μg/ml), *E. faecium* (MIC = 12.5 μg/ml), and *S. aureus* (MIC = 3.2 μg/ml) (Porter et al., 2000). Subsequently, they synthesized a series of 20-unit oligomers with varying cationic residues from 100 to 30% to investigate the structure-activity relationships. When the cationic residues decreased to 60% in the whole molecule, the oligomer showed comparable activity with magainin II against a panel of bacteria, including *E. coli* (MIC = 12.5 μg/ml), *B. subtilis* (MIC = 3.1 μg/ml), *E. faecium* (MIC = 12.5 μg/ml), *S. aureus* (MIC = 25 μg/ml) (Mowery et al., 2007). Importantly, it showed significant improvement of biocompatibility to erythrocytes compared to magainin II, with a minimum hemolytic concentration (MHC) of 100 μg/mL. Thus, they concluded that the best proportion of cationic residue was 63%, achieving a selectivity index (MHC/MIC) of 32. Recently Chan-Park et al. reported two types of glycosylated cationic block co-beta-peptides that showed excellent *in vivo* antimicrobial performance. PDGu(7)-b-PBLK(13) showed directly killing effect against Gram-positive bacteria MRSA USA 300 in the *in vivo* biofilm wound infection model, and PAS8-b-PDM12 could sensitize a variety of antibiotics to recuse the mice from a lethal systemic infection caused by all the ESKAPE Gram-negative bacteria (Zhang et al., 2019; Si et al., 2020).

Although synthetic cationic polymers have shown great potential in antimicrobial applications, they are suffered from some disadvantages which limit their applications in biomedical fields. First, amphiphilic cationic and hydrophobic polymers may cause hemolysis due to the non-specifically hydrophobic interaction with the erythrocyte membrane. Second, the physiological stability of AMPs and their memetics is poor because most of them are composed of natural amino acids. Third, polypeptides with a specific sequence are till now hard to manufacture industrially (Hancock and Sahl, 2006). Currently, only a few AMP-related drugs such as colistin and daptomycin have been approved by the United States FDA to use in clinic (Eckert, 2011). All these antibacterial materials were summarized in Table 1.

**TABLE 1:** Classification and property of antibacterial materials.

Classification	Sub-class	Antibacterial capability			References
		
		Gram-negative	Gram-positive	Fungus	
Cationic polymers	PPy derivatives	EC, AB, BS, EC, KP, PA, ST	CR, SA		Li and Shen, 2000; Allison et al., 2007; Sellenet et al., 2007
	Poly ionic liquids	AB, EC, ECL, PA, KP	SA, VRE	AN, CA	Liu et al., 2012, 2013; Guo et al., 2018; Yuan et al., 2019
	Guanidine salts	AB, EC, KP, PA	SA		Chin et al., 2018
	COEs	AB, EC, KP	EF, SA		Liu et al., 2012; Bazan et al., 2018
	Dendritic polyethylene imine (PEI)	BS, EC, PA, SMA	EF, SA, SE, SM, SS	CA	Paula et al., 2008; Davide et al., 2013
	Antimicrobial peptides (AMPs)	AB, EC, HP	CR, SA	CA	Eckert, 2011; Li et al., 2012; Lam et al., 2016; Hou et al., 2017; Pranantyo et al., 2017; Xu et al., 2019
	β-peptide polymers	AB, BS, EC, KP, PA	EF, SA		Porter et al., 2000; Mowery et al., 2007; Si et al., 2020
Antibacterial hydrogels	Polymeric hydrogel	AB, BS, EC, KP, PA, ST	CR, SA		Bai et al., 2016; Baral et al., 2016; Hoque et al., 2017; Wang et al., 2017
Hydrogels containing antibacterial components	Antibiotic-containing	EC, PA	SA		Das and Pal, 2015; Gustafson et al., 2016; Hanna and Saad, 2019
	NP-containing	EC	SA		Sondi and Salopek-Sondi, 2004; Shahverdi et al., 2007; Wahid et al., 2015, 2017

*AB, *Acinetobacter baumannii*; AC, *Amphora coffeaeformis*; AN, *Aspergillus niger*; BS, *Bacillus subtilis*; CA, *Candida albicans*; CR, *Chlorella regularis*; EC, *Escherichia coli*; ECL, *Enterobacter cloacae;* EF, *Enterococcus faecalis*; HP, *Helicobacter pylori*; KP, *Klebsiella pneumoniae*; PA, *Pseudomonas aeruginosa*; SA, *Staphylococcus aureus*; SE, *Staphylococcus epidermidis*; SM, *Streptococcus mutans*; SMA, *Stenotrophomonas maltophilia*; SS, *Streptococcus salivarius*; ST, *Salmonella typhimurium*; VRE, *Vancomycin-resistant enterococcus*.*

### Antibacterial Hydrogels

The hydrogel is a super-hydrophilic polymeric material with a 3D network. The polymeric hydrogel with intrinsic antibacterial activity possesses both super-hydrophilicity and good antibacterial effects in nature. Hydrogel poly(N-acryloyl glycine), formed from N-acryloyl glycinamide and 1-vinyl-1,2,4-triazole via hydrogen bonding interaction of two amide groups in the side chain of N-acryloyl glycinamide, has good bactericidal activity. Moreover, it possesses the good capability of self-healing, thermoplastic, and re-shape at low temperatures due to the group of 1-vinyl-1,2,4-triazole in the molecule. It can be used as matrixes of 3D printing or injectable biomaterials. The results of cytotoxicity tests and *in vivo* implantation confirmed that this hydrogel has good biocompatibility (Wang et al., 2017). Hydrogels, N-(2-hydroxypropyl)-3-trimethyl chitosan chloride (HTCC) and the bioadhesive poly(dextran aldehyde), not only are effective against Gram-positive and Gram-negative bacteria, but also promote the wound healing in rats’ model (Hoque et al., 2017). Whilst, a hydrogel made from dipeptides-containing long-chain amino acids has bactericidal activity against Gram-negative *E. coli* and *P. aeruginosa* but displays a weak effect on Gram-positive bacteria (Baral et al., 2016). Enzymatic A9K2 hydrogel containing fetal bovine serum (FBS) or plasma amine oxidase (PAO) was once synthesized. The experimental results verified its biocompatibility and effectiveness against Gram-positive and Gram-negative bacteria, including *P. aeruginosa*, *S. aureus*, and *B. subtilis* (Bai et al., 2016).

### Hydrogels Containing Antibacterial Components

Hydrogel is often used as organic carrier in the field of biomedicine due to its excellent biocompatibility and substance transferring capabilities. The molecular structure, mechanical and rheological properties can be tuned by changing temperature, pH value, ionic strength, and ultraviolet treatment, *etc*. The drug release profiles can be adjusted to meet multiple needs (Ng et al., 2014; Yang K. et al., 2018). Antibacterial hydrogels are mainly divided into two types: hydrogels containing antibiotics, and metal nanoparticles, according to the encapsulated ingredients (Li et al., 2018).

Antibiotic-containing hydrogel refers to the one in which antibiotic drugs are mixed or embedded in the hydrogel molecular network. When antibiotic-containing hydrogel is coated onto the infected site, the antibiotic in the hydrogel will be gradually released to achieve the bactericidal effect. This kind of treatment minimized the amount of antibiotics, thus might reduce the side effects of the drug compared with oral administration. At the same time, the hydrogel matrix is helpful to keep the wound moist, which is conducive to wound healing. Polyethylene glycol fumarate/sodium methacrylate (PEGF/SMA) hydrogel was used to treat bacterial infections, in which vancomycin was loaded through electrostatic interaction (Gustafson et al., 2016). A continue release up to 4 days was achieved at the surgical site, which helped to restrain methicillin-resistant *S. aureus* and promoted wound healing. pH responsible poly (2-hydroxyethyl methacrylate)-based crosslinked hydrogel (pHEMA) was used as hydrogellic carrier (Das and Pal, 2015). N-trimethyl chitosan/carboxymethyl xanthate (TMC/CMXG) hydrogel manufactured from chitosan, dimethyl sulfate (DMS), and methylol xanthan gum (CMXG) is a good matrix to continuously release ciprofloxacin at a constant rate with non-cytotoxicity to mammalian cells (Hanna and Saad, 2019).

The second type is metal nanoparticle (NP)-containing hydrogels, where metal nanoparticles were embedded in the molecular network of hydrogels. It is well-known that metal NPs are easily oxidized, moisturized, and agglomerated in the air. All these shortcomings will be avoided after they are loaded in the hydrogel matrix. The most commonly used metals are silver (Ag) and zinc oxide (ZnO) NPs, because these two metals can directly enter the bacterial cells to bind the DNA or oxidative metabolic enzymes, leading to hinder the bacteria metabolism or mutate the DNA. Both the hydrogels are good bactericidal materials against *E. coli* and *S. aureus* (Shafagh et al., 2018; Neacsu et al., 2019). Crosslinked carboxymethyl chitosan (CMCh)/ZnO hydrogel, produced from *in situ* formation of ZnO nanorods in the crosslinked carboxymethyl chitosan (CMCh) matrix, is effective against *E. coli* and *S. aureus* (Wahid et al., 2015). The hydrogel CMCh/CuO displayed a similar bactericidal effect on *E. coli* and *S. aureus* when nanoparticle CuO was used instead of ZnO in the hydrogel (Wahid et al., 2017).

## Preparation and Application of Antibacterial Coating

Medical devices or implants are often surrounded by cells or extracellular matrix like proteins and polysaccharides, which are conducive to bacteria colonization and biofilm formation, further enhancing the tolerance of bacteria to antibiotic treatment. Thus, it is necessary to change the physical and chemical characteristics of the device surface to prevent pathogen infection. The cationic polymers and hydrogels stated as above can be used as building materials to construct the coatings on various medical devices to achieve the bacteria resistance. Anti-infective surfaces mainly include: (1) anti-adhesive surfaces; (2) surfaces with contact antibacterial properties; (3) surfaces with a combination of anti-adhesion and contact sterilization. Generally, hydrophilicity is unfavorable for microorganism adhesion. Therefore, many studies focus on improving the surface hydrophilicity, for example, fixing polysaccharides, such as hyaluronic acid and heparin, or hydrophilic polyethylene glycol (PEG) onto device surface to obtain a significantly hydrophilic surface or interface (Busscher et al., 2012; Junter et al., 2016). On the other hand, grafting with antimicrobial reagents on the surface may realize the contact sterilization (Konai et al., 2018). Expectably, the devices will obtain the excellent bactericidal surface if they are treated by anti-adhesion and bactericidal reagent grafting with the optimized components.

There are various methods for grafting antibacterial agents on the device surface. Generally, it can be divided into three categories, namely covalent grafting, material blending, and layer-by-layer (LBL) assembly. The covalent grafting method is to form a stable modified layer with covalent bonds between the graft molecule and the device surface; the material blending and LBL assembly use non-covalent bond reactions, such as electrostatic attraction or hydrogen bonding. The later surfaces will not be as stable as the former, covalent grafting. Sometimes, cross-linking agents will be used to cross-link the blended or LBL assembled materials to enhance their stability.

### Covalent Grafting

There are two categories in the covalent grafting method, “grafting to” and “grafting from.” The method of “grafting to” refers to covalently grafting a polymeric chain via its reactive functional groups onto a device surface material through chemical reactions. “Grafting from” is the method in which the target monomers were polymerized through the initiative groups that were generated beforehand on the surface substrate. The method of “grafting from” is more often used because a larger amount and higher density of monomers can be grafted, compared with the method of “grafting to” (Steve et al., 2003). However, these two methods were not clearly classified in surface modification on devices.

Polyethyleneglycol (PEG) based materials possess many desired properties like hydrophilicity, lubricity, moisture retention and compatibility, *etc.*, and thus have been widely applied in cosmetics, pharmaceutical, pesticide and food package. For example, a bactericidal and non-thrombogenic small diameter vascular graft was manufactured via 3D-printing technology with PEG-polycaprolactone (PCL)-S-Nitroso-N-acetyl-D-penicillamine (SNAP) matrix. This graft was verified to have antibacterial efficacy against both Gram-positive and Gram-negative bacteria, as well as antithrombogenic properties in plasma (Kabirian et al., 2019). Polycarbonate was prepared with cations, PEG and dopamine at the side terminus, followed by surface modification on silicon substrate, which was treated in trimethylolaminomethane for 24 h at 70°C, through cross-linking reaction between amine from the substrate and dopamine from polycarbonate (Chuan et al., 2015). This modification made the substrate obtain the properties of both antimicrobial and antifouling properties due to the functional components of PEG and cationic quaternary ammonium. For example, it maintains the antibacterial effect against *S. aureus* for 14 days. Meanwhile, it can prevent protein and platelet adsorbing on the surface due to the super-hydrophilic PEG, which is conducive to reduce thrombosis occurrence. Another PEG containing polymer, methacrylate (MA)-PEGn-b-antimicrobial polypeptide copolymer, was synthesized from N-carboxyanhydride and methacrylate-PEGn-tosyl, in Ma group (Gao et al., 2017). This diblock amphiphilic copolymer has properties of antimicrobial, antifouling, and antibiofilm. The surface obtained excellent antimicrobial activity against several pathogenic bacteria (*E. coli, P. aeruginosa*, and *S. aureus*), and effectively prevent biofilm formation after the copolymer was grafted onto silicone rubber (commonly used catheter material) via plasma/UV-induced surface polymerizations. This bottlebrush coating also greatly reduced protein adsorption and platelet adhesion, indicating its excellent antifouling ability and blood compatibility.

Monomers or polymers containing double bonds in their molecular structures can be photo-initiated in the presence of a radical initiator. They are thus often used for grafting purposes. Three polyoxo norbornene-based polyzwitterions were fabricated through UV activation and thiol-ene click reaction. A silane-treated substrate followed by polynorbornene grafting exhibited strong protein repellent and biofilm inhibition (Kurowska et al., 2017). Moreover, this substrate is friendly to red blood cells and mucosal keratinocytes under the proper ratio of zwitterions and polynorbornene. This technology can be used on various substrates with active radical sites after silane or benzophenone treatment. Therefore, it has the versatility and is expected to be widely applied to various medical devices. A semi-interpenetrating polymer network (SIPN) was formed from quaternized poly [2-(dimethylamino) ethyl methacrylate-methyl methacrylate] [poly (DMAEMA-co-MMA)], and polymerized ethylene glycol dimethacrylate. This SIPN polymer was coated on contact lens to produce anti-fog/antimicrobial dual-functional surface. The anti-fogging function comes from the hydrophilic/hydrophobic balanced smooth coating, and antimicrobial from the quaternized structure, which can completely kill Gram-positive *Staphylococcus epidermidis* and Gram-negative *E. coli*. This dual function polymer is expected to have a broad application in contact lenses (Zhao et al., 2016).

“Polymer brush” is an imaginable term to describe an architecture in which polymer chains are terminally tethered to a surface at a very high density. This grafting from the polymerization approach is a good way to grow the chains directly on the substrate surface. Monomer [2-(methacryloyloxy) ethyl]-trimethylammonium chloride (META), N-(3-sulfopropyl)-N-(methacryloyloxyethyl)-N, N-dimethylbetaine (SBMA) and 2-methacryloyloxyoxyethyl phosphate choline (MPC) are polymerized to form a brush-like polymer and grafted on a substrate through surface-initiated atom transfer radical polymerization. The positively charged META functionalized the surface with antimicrobial activity, while the zwitterionic SBMA and MPC make the surface antifouling and resistant to bacterial adhesion. This technology provides a potential method to establish antifouling and antimicrobial surfaces on medical devices (Xu et al., 2017). Similarly, quaternary ammonium methacrylate compound (QAC-2) had a perfluoroalkyl tail group at one end and an acrylic moiety at the other end. A one-step UV curing of QAC-2 and methyl methacrylate (MMA) using ethylene glycol dimethacrylate (EGDMA) as a cross-linking agent obtained a polymer-coated surface on a glass slide which had been treated by rotenone solution and produced active hydroxyl groups. This modified glass slide possessed excellent antimicrobial properties (Jie et al., 2015). Methacrylate was initiated to polymerize on quartz glass and titanium (Ti) surfaces containing silane groups. Using this polymeric molecule as the anchor, the peptide was further coupled to finally offer quartz glass and Ti surface excellent antimicrobial activity (Yu et al., 2015). Xu et al. (2017) grafted L-arginine methyl ester-methacrylamide (Arg-Est) and L-arginine-methyl Acrylamide (Arg-Me) polymer onto glass slides using surface-induced reversible addition-fragmentation chain transfer polymerization (RAFT). Compared with purely Arg-Est grafted surface, it (Arg-Est-Me) has better antimicrobial properties. Unexpectedly, this antimicrobial surface will shift to the antifouling surface after the grafted Arg-Est-Me is hydrolyzed; all bovine serum albumin, Gram-positive *S. aureus*, Gram-negative *E. coli*, and *Amphora coffeaeformis* were resisted to adhesion. The *in vivo* tests verified that the grafted Arg-Est-Me component has good biocompatibility and stability.

Poly-N-isopropylacrylamide (poly-NIPAAM) has been widely used in tissue engineering and micro-structure in micro-fluidic applications. It was used to combine with quaternary ammonium salt [2-(dimethylamino)-formaldehyde ethyl acrylate (DMAEMA)] and grafted onto glass, polydimethylsiloxane (PDMS), and silicon wafer substrates using RAFT method (Wang et al., 2016). When the temperature increased, the component NIPAA of the copolymer brush became hydrophobic, which helped the surface trap microorganisms and the surrounding DMAEMA component contributed to killing the trapped bacteria. The bactericidal efficiency was up to reduce three logarithmic levels of *S. aureus* and two logarithmic levels of *E. coli*. NIPAA became hydrophilic when the temperature decreased, which oppositely helped to release the killed microorganisms together with the coated surface. This capability makes the surface switchable between bactericidal and antifouling even after four deposition/release cycles.

Dopamine with the properties of fast oxidizing and self-polymerizing in water has been widely applied for surface modification on a variety of substrates (Qiu et al., 2018; Palladino et al., 2019). For example, acrylate or acrylamide copolymer can be covalently bonded onto polydopamine (PDA) using Michael addition, through which these copolymers were coated onto substrates, or one-step co-deposition of dopamine and methacrylate (MA), both provided the substrates excellent antimicrobial, antifouling, and biocompatible properties (Liu and Huang, 2016; Zhang et al., 2017). A rough PDA coating (rPDA) on substrates like glass, stainless steel, plastic, and gauze was verified to display excellent antibacterial effect against Gram-positive *S. aureus* and Gram-negative *E. coli* and *P. aeruginosa* (Su et al., 2016). Indispensable catheters are often used in clinic, but catheter-associated infection and biofilm-forming are significant clinical problems. Chan-Park group prepared a silicon catheter with properties of non-leachable antibacteria and antibiofilm, in which PDA containing multi-density of α-bromoisobutyryl bromide (BIBB) initiators was firstly bond on the catheter, followed by atom-transfer radical polymerization of cationic (3-acrylamidopropyl) trimethylammonium chloride (AMPTMA) or quaternized polyethylenimine methacrylate (Q-PEI-MA) together with a cross-linker (polyethylene glycol dimethacrylate, PEGDMA). This catheter exhibited the capability to prevent bacterial colonization, reducing 1.95 and 1.26 logarithmic levels of methicillin-resistant *S. aureus* and VRE, respectively, in mouse urethral infection models (Zhou et al., 2017).

Chitosan and its derivatives have been widely studied and applied due to their excellent properties like biocompatibility and antibacterial activity (Sahariah and Másson, 2017). Ti substrate was firstly treated with alkali to form a nanoscale topology under heating, on which dopamine was self-polymerized followed by incorporation of hydroxyapatite (HA) and carboxymethyl chitosan (CMCS) (Yang M. et al., 2018). HA can promote the adhesion of soft tissues while CMCS can work as an antibacterial effect. Moreover, both HA and CMCS are helpful for the adhesion and proliferation of human gingival fibroblasts. The Ti substrate was also modified by chitosan via coupling agent of triethoxysilylpropylsuccinic anhydride, and achieved the antibacterial effectiveness against *E. coli* and *S. aureus* (D’Almeida et al., 2018). Another example of chitosan-based coating on polyurethane (PU), polyvinyl fluoride (PVF) and Ti substrates was provided by Neoh group (Li et al., 2017). They treated the substrates with oxygen plasma to introduce hydroxyl groups on the surface, through which dicaprolactone, agarose (AG) and quaternized chitosan (QCS) were covalently bonded via oxidative conjugation of thiol and hydroxyl group under UV irradiation. The component AG reduced the biofilm formation of *P. aeruginosa* and *S. aureus* by more than 99%, while the QCS reduced the bacterial contamination by more than 95%.

Polyhexamethylene biguanide is a class of antimicrobial polymers with broad-spectrum antibacterial efficacy against both Gram-positive and Gram-negative bacteria because of its strong positive potential in the molecule. However, this positive potential makes it toxic to tissues/cells. Moreover, the poisonous effects will have to be weighed if leaching out to the environment. Therefore, PHMB is not yet applied in a variety of industries. Several methods were studied to modify the PHMB molecule. For example, PHMB was reacted with allyl PEG to form PEG-b-PHMB, which was grafted onto argon plasma treated silicone resin to give the surface anti-adhesive and antimicrobial properties (Zhi et al., 2017). PHMB can effectively kill bacteria while hydrophilic PEG can prevent bacteria fragments from forming biofilm to ensure a continuous antibacterial effect on the surface. In the *in vivo* tests with rat infection model, it has been shown that the *E. coli* was reduced for five logarithms in rat subcutaneous implantation. More importantly, the introduction of PEG reduced the toxicity of PHMB to mammalian cells, which is expected to be applied and developed in the surface coating of medical equipment (Su et al., 2017).

The recent study about surface modification mainly focuses on the innovation of responsive polymer brushes that are responsive to factors like humidity, temperature, pH, *etc*., in the solution (Chen et al., 2010). Hu et al. (2013) studied polymethacrylate sulfonate betaine (pSBMA) and polymethacrylate carboxylate betaine (pCBMA) as silver-embedded substrates. The product, pCBMA-silver hybrid (CB-Ag), kills bacteria by contact and releases dead bacteria under humid conditions. Poly(N-isopropyl-acrylamide) (PNIPAAm) is a typical thermally responsive polymer (Yaghoubi and Parsa, 2019). Its solubility responds to reversible changes of temperature with a critical temperature of 32°C; Poly(methacrylic acid) (PMAA) is a typical pH-responsive polymer with a large number of carboxyl groups (COOH) on its repeating unit (Qu et al., 2015). The PMAA chain shows a collapsed conformation in an acidic aqueous solution and a swollen conformation in an alkaline aqueous solution. Yan et al. (2016) used the outer layer of pH-responsive poly(methacrylic acid) (PMAA) as a regulator, while the AMP was covalently fixed on the inner layer. The PMAA hydration layer makes the layered surface resistant to initial bacterial adhesion and biocompatible under physiological conditions. When colonize the surface, the bacteria trigger a pH drop that folds the outermost PMAA chain, thus exposing the potentially bactericidal AMP to kill bacteria on demand. When the environmental pH increases, the PMAA chain restores hydrophilicity. The materials, properties, and applications of covalent grafting method were summarized in Table 2.

**TABLE 2:** Materials, properties, and applications of covalent grafting method.

Materials	Property	Substrate example	References
PEG-based polymer	Antifouling, antibacterial, antibiofilm, reduce thrombosis	Silicone rubber	Chuan et al., 2015; Gao et al., 2017; Kabirian et al., 2019
Double bond- containing polymer	Antifouling, antimicrobial, anti-fogging	Contact lens	Zhao et al., 2016; Kurowska et al., 2017
Polymer brush	Antifouling, antimicrobial	Glass, quartz, Ti	Jie et al., 2015; Yu et al., 2015; Xu et al., 2017
Poly(NIPAAM)	Antifouling, bactericidal	Glass, PDMS, silicon	Wang et al., 2016
PDA	Antifouling, antimicrobial, antibacterial, antibiofilm	Glass, stainless steel, plastic, gauze, catheter	Liu and Huang, 2016; Su et al., 2016; Zhang et al., 2017; Zhou et al., 2017
Chitosan and derivatives	Antibacterial, antibiofilm	Ti, PU, PVF	Li et al., 2017; D’Almeida et al., 2018; Yang M. et al., 2018
PHMB	Broad-spectrum antimicrobial, bactericidal, antifouling, antibiofilm	Silicone resin	Su et al., 2017; Zhi et al., 2017
Responsive polymer brush	Antifouling, antimicrobial, antibacterial	Glass, Si, PU, MWCNTs, SiO_2_, Fe_3_O_4_	Chen et al., 2010; Hu et al., 2013; Qu et al., 2015; Yan et al., 2016; Yaghoubi and Parsa, 2019

### Layer-by-layer Technology

The technology of LBL assembly refers to the adsorption of electrolyte or complementary compounds on the substrate surface LBL, till the expected density or amount was achieved. This method is simple to operate and has no requirements for the substrate shape. The thickness and properties of the adsorption layer can be adjusted at the molecular scale. The simplicity and versatility of LBL technology attracted abroad interests and has much studied to apply in many fields, particularly in applications on medical devices with various matrix and shapes (Gentile et al., 2015; Shi et al., 2017). For example, it was used to modify silicon substrate with polyacrylic acid (PAA, as the polyanion) and polyetherimide (PEI, as the polycation) to obtain a multilayer PAA/PEI assembled film (Sze Yinn et al., 2012). After modification, the silicon substrate exhibited strong antifouling performance and antimicrobial activity. It will enhance the antifouling if the polyanions exposed (as the outermost layer) and increase the hydrophobicity (more sensitive to protein adsorption) if the longer alkyl chain in PEI is explored.

Chitosan and heparin, an antagonistic compound pair, were applied to modify the coronary stent with LBL technology, where chitosan is positively charged and heparin is negatively charged. After treatment, the modified stent obtained better anticoagulant performance, compared with the primitive stent. It also enhanced the regeneration of vascular endothelial cells (Meng et al., 2009). Polydimethylaminoethyl methacrylate (polycation) and cellobiose dehydrogenase (CDH, polyanion) or polybenzenesulfonic salt (polyanion) were another antagonistic pair. Three LBL layers on polydimethylsiloxane (PDMS) catheters were assembled to obtain a medical surface with excellent antifouling and antimicrobial properties (Vaterrodt et al., 2016). The enzymatic activity of CDH can be adjusted from the depth of embedding in the multilayer and the chemistry of the polymers in the pair.

Chitosan modified by tannic acid and small AMP were assembled LBL on stainless steel plates (Pranantyo et al., 2018). The obtained surface exhibited not only antibacterial activity against *E. coli* and *S. aureus*, but also good biocompatibility to primary fibroblasts. Polyethylene terephthalate substrate (PETG, commonly used in the orthodontic appliance) was treated by oxygen plasma to activate the surface, followed by LBL deposition of carboxymethyl cellulose (CMC) and chitosan (Park et al., 2018). The modified PETG with good chemical and physical stability was tested to be super-hydrophilic on the surface. It is able to prevent bacterial adhesion, reduce the bacteria number as high as 75%. The LBL multilayer film of branched PEI and urushiol on various substrates like silicon wafer, PDMS, and glass slide in an acidic solution was found to have antimicrobial activity with the enhanced hydrophobicity and mechanical stability due to the presence of urushiol component (Hyejoong et al., 2015).

In summary, LBL assembly is a simple and universal technique for making antimicrobial coatings on medical devices. The properties of the modified surface can be adjusted by changing polycations and polyanions, the number of multilayers, the thickness of the assembled film, and the chemical composition of the exposed layer. In addition, ingredients can be added into the LBL film to obtain some special functions to meet the requirements of medical devices (Gessner and Vilesov, 2019). It is expectable that the LBL assembly will be a good technology for industrially manufacturing medical devices with promising antimicrobial features in the near future.

### Surface Polymer Blending and Other Methods

Surface polymer blending is to mix different polymers without changing their respective properties on the device surface. The final features of the surface can be regulated by changing the objective components or the ratios to meet various requirements of medical equipment. For example, polyhexamethyleneguanidine dodecylbenzenesulfonate (PHMG-DBS) was coated on silicon tube, which was used as a catheter or tracheal intubation. Tested with the method of the international standard (ISO 22196), the results showed that the bacterial counts on the tube were reduced by more than three logarithmic scales. Little leaching of the polymers was detected in water or saline, indicating that the modified layer was quite stable (Ghamrawi et al., 2017). The polylactic acid (PLA) scaffold displayed the bactericidal capability if PHMG was mixed into PLA material. Meanwhile, the biocompatibility of the scaffold from the biodegradable PLA bulk was maintained (Llorens et al., 2015). A polyurethane (PU) catheter modified with poly (diallyldimethylammonium chloride) (pDADMAC) exhibited good antibacterial performance. It reduced the possibility of biofilm formation for eight logarithmic levels against *S. aureus* and one logarithmic level against *P. aeruginosa*, compared with PU plain tube (Dirain et al., 2016). Besides, pDADMAC-PU tube displayed good antimicrobial activity in blood plasma. Therefore, it is hopeful to be used as implantable catheters. Quaternized chitosan was added into denture powder during the composite dentures production. This denture was tested to be biocompatible to rat fibroblasts. On the other hand, the quaternized chitosan assisted the dentures to possess the capabilities of corrosion resistance and antibacterial activity, which might provide a promising material candidate for oral denture (Song et al., 2016).

Contact active antimicrobial films were prepared by simply blending cationic amphiphilic block copolymers with commercial polystyrene (PS), where the copolymers were synthesized from 4-{1-[2-(4-methylthiazol-5-yl)ethyl]-1H-1,2,3-triazol-4-yl} butyl methacrylate (TTBM) and contained antimicrobial block bearing flexible side chain. This blended film exhibited excellent antimicrobial activities against Gram-positive, Gram-negative bacteria and fungi. When the copolymer ingredient was up to 50 wt%, a more than 99.999% killing efficiency against the above microorganisms was achieved. Remarkably, the film containing 50 wt% copolymer presented better antimicrobial activity than the films prepared exclusively from the cationic copolymers, which implies a reduction of antimicrobial agents and might provide new insights for the better designing of antimicrobial coatings (Cuervo-Rodriguez et al., 2017). The composite polymers blending of Pluronic F-127, F-127-AMP, and F-127-RGD (arginine-glycine-aspartic acid) with appropriate proportions were coated on silicone rubber. The resulting material showed the antiadhesive and antibacterial properties against *S. aureus, S. epidermidis*, and *P. aeruginosa* due to the “brush” topography of the blending polymers while RGD peptides enhanced the adhesion and spreading of human fibroblast cells. This technology is anticipated to be used as an infection-resistant coating for medical implants and devices (Muszanska et al., 2014).

Our group has investigated a novel antimicrobial coating technology, in which the silicon substrate was modified by a cross-linked polyvinylpyrrolidone (PVP) under vacuum, immediately followed by *in situ* grafting of PVP homopolymers using solvent-free graft-from polymerization method. A total of 99.9% antifouling enhancement was achieved after the catheter was modified while the improved biocompatibility was also observed in a 4-week *in vivo* tests in mice because of good hydrophilicity of PVP (Sun et al., 2019). Dimethylaminomethylstyrene (DMAMS) or poly (dimethylaminomethylstyrene-co-ethylene glycol diacrylate) [P(DMAMS-co-EGDA)] at different crosslinking ratios was once vaporously deposited on flat and porous substrates, using tert-butyl peroxide (TBP) as an initiator and ethylene glycol diacrylate (EGDA) as a cross-linking agent. A total of 99.99% reduction of bacteria adhesion against *B. subtilis* and *E. coli* was achieved with insignificant leaching within experiment interval (Ye et al., 2010, 2011).

## Conclusion and Perspective

The antimicrobial coatings are conducive to overcome pathogenic infections in the hospital environment, and provide some specific functions for medical devices. The coated polymers with large molecular weight are superior to those with small molecules in many aspects, such as better stability, less leaching and more functionalized via chemical and physical designs. Particularly, less leaching will be helpful to reduce the poisonous effects on the human body or the pollution on the natural environment.

In this article, we reviewed the mechanism and advantages of antimicrobial polymers, and the potential applications in medical device modifications. There are some technologies to be explored according to the chemistry, appearance and micro-structure of the targeted substrates and equipment. The covalent grafting method allows the connection by covalent bonds between the modification materials and the substrate surface, which can form a surface coating with excellent stability and less leaching; The LBL assembly makes the surface modification easy to achieve. In addition, the stability of the modified layer can be improved by cross-linking reaction while non-covalent connections of polymer blending would give more material choices. These methods have their advantages and can be selected as required.

At present, there are still some challenges for the applications of antimicrobial polymer coatings. Firstly, the mechanism, efficiency, and the relationship between them are not studied clearly, especially at the molecular level. Some functional devices already used in clinic are not perfect, particularly for those implanted in the body for a long time or needed to be reusable. Many antimicrobial medical devices lack adequate *in vivo* studies and clinically attempts. The results of studies focusing on protein or cell levels are not entirely suitable for the much complex body’s environment, where the biocompatibility of the devices is sometimes more important than the functionality. In short, medical devices modified with antimicrobial polymer coatings are expected to be used, instead of plain metal devices, to promote the devices’ functionality in the future.

## Author Contributions

HQ and ZS: writing the manuscript. YZ and MC-P: correcting and proof. YL, PF, XW, WH, and LX: collecting and filing up the references. DH: submitting information of medical devices. All authors contributed to the article and approved the submitted version.

## Conflict of Interest

DH was employed by the company Ningbo Baoting Biotechnology Co. The remaining authors declare that the research was conducted in the absence of any commercial or financial relationships that could be construed as a potential conflict of interest.
